# Notes by a Surgeon on Active Service

**Published:** 1915-07-03

**Authors:** 


### NOTES BY A SURGEON ON ACTIVE SERVICE.

### Applications to Open Wounds.

Iodine solution is apt to decompose, and is always objectionable on account of its staining and fuming properties. Convenient and equally useful substitutes are a 2-per-cent. solution of picric acid, a 10-per-cent. solution of salicylic acid, or a 20-per-cent. alcoholic solution of methyl salicylate. These do not smart when applied, and are reliable in their antiseptic properties.

### Morphia Injections.

It is often difficult, on the field, to obtain water for dissolving the ordinary tablets for hypodermic use carried in the dressing-case. In such circumstances the tablet may be put under the tongue of the wounded man—the so-called sublingual medication. A better expedient is to carry a small pocket bottle of morphia solution with an indiarubber capsule over the mouth, similar to the bottles used for inoculation purposes. The needle of the syringe can be plunged through the indiarubber, making a valvular opening that readily shrinks. I use omnopon solution, one grain in twelve minims, to which dose one-hundredth of a grain of atropine has been added, and I have found this solution most efficacious in the field.

### Thirst on the March.

Smokers suffer more from thirst than any other details, and smoking should therefore be discouraged when water supplies are scarce or polluted. Cold weak tea carried in the water-bottles is perhaps the best thirst quencher, but training is undoubtedly the best prophylactic. A man can be trained to go for several hours without water, or to content himself with occasional wetting of the lips and palate. Where water is suspicious or unobtainable, lime-juice lozenges or gum jujubes flavoured with some sour-tasting fruit juice are the best preventative of thirst. These should be handed out to the men on the march, with instructions to chew one occasionally when the thirst becomes severe.

### Enteric Inoculation After-effects.

My experience goes to show that some of these effects can reasonably be ascribed to bad technique. If the needle pricks a vein, for instance, the after-effects are more often complained of than when the injection has been made subcutaneously. Too vigorous application of iodine, too, appears to promote the rapid absorption of the vaccine and to encourage anaphylaxis.

